# Evidence-Based Interventions and Screening Recommendations for Colorectal Cancer in Comprehensive Cancer Control Plans: A Content Analysis

**Published:** 2009-09-15

**Authors:** Julie S. Townsend, Lisa C Richardson, C. Brooke Steele, Dana E White

**Affiliations:** Division of Cancer Prevention and Control, National Center for Chronic Disease Prevention and Health Promotion, Centers for Disease Control and Prevention; Centers for Disease Control and Prevention, Atlanta, Georgia; Centers for Disease Control and Prevention, Atlanta, Georgia; Centers for Disease Control and Prevention, Atlanta, Georgia

## Abstract

**Introduction:**

Colorectal cancer is the third most commonly diagnosed cancer and third leading cause of cancer death in the United States. The extent to which Comprehensive Cancer Control (CCC) programs in states, tribal governments and organizations, territories, and Pacific Island jurisdictions address evidence-based recommendations and interventions for colorectal cancer in their CCC plans is largely unknown.

**Methods:**

We downloaded CCC plans posted on the Cancer Control PLANET Web site for review. We searched the plans for key terms, identifying potential evidence-based content surrounding colorectal cancer prevention and early detection. Content was abstracted for further review and classification.

**Results:**

Of 55 plans reviewed, 54 (98%) referred to evidence-based recommendations or interventions for colorectal cancer or indicated they intended to refer to the evidence base when developing programs. More than 57% (n = 31) of programs referred to the American Cancer Society guidelines, 41% (n = 22) referred to the United States Preventive Services Task Force, and 11% (n = 6) referred to the *Guide to* *Community Preventive Services*. Few programs mentioned Research Tested Intervention Programs (n = 1), National Cancer Institute's Physician Data Query (n = 4), Cochrane Reviews (n = 2), or Put Prevention Into Practice (n = 2) in reference to evidence-based interventions for colorectal cancer prevention.

**Conclusion:**

Most CCC programs discussed either evidence-based screening guidelines or interventions in their cancer plans, although many mentioned this information exclusively as background information. We recommend that program planners be trained to locate evidence-based interventions and use consistent common language to describe them in their plans. CCC program planners should be encouraged to conduct and publish intervention studies.

## Introduction

Colorectal cancer (CRC) is one of the most commonly diagnosed cancers in the United States. In 2004, more than 145,000 people — approximately 74,000 men and approximately 71,000 women — were diagnosed with CRC, making it the third most common cancer in men and women (1). Although CRC affects both sexes and all races, the incidence is disproportionately high in men (1), African Americans (1), and American Indians/Alaska Natives living in the northern and southern plains and Alaska (2,3).

Regular screening for CRC can reduce incidence by 76% to 90% (4) and deaths by as much as 60% (5). Even though clinical evidence from randomized controlled trials shows that screening for CRC decreases the incidence of and deaths from CRC (4,6), new reports indicate that only 50% of Americans are screened (7) and that prevalence varies from 52% to 71% among states (8). Comprehensive Cancer Control (CCC) programs in all 50 states, the District of Columbia, and various tribal governments, territories, and jurisdictions are challenged to find interventions to raise CRC screening rates. CCC programs use "an integrated and coordinated approach to reducing cancer incidence, morbidity, and mortality through prevention, early detection, treatment, rehabilitation, and palliation" (9).

In 1998, the Centers for Disease Control and Prevention (CDC) provided funding to 5 states and 1 tribal health board that had existing CCC plans (10). Since 1998, the number of programs funded by CDC through the National Comprehensive Cancer Control Program (NCCCP) has grown from 6 to 65 (www.cdc.gov/Cancer/ncccp/). Health agencies use the funding to establish broad-based CCC coalitions, assess the burden of cancer, and develop and implement CCC plans. These plans include interventions to reduce cancer incidence and mortality (10).

Evidence-based public health has its roots in evidence-based medicine (11) and arose as a need for evidence-based decision making in public health (12,13). The Institute of Medicine report *The Future of the Public's Health in the 21st Century* (14) proposed that, to have an effective public health system, evidence should be translated into practice and be the foundation of decision making and the measure of success.

Using evidence-based interventions allows for resources to be allocated effectively because interventions work in  given populations, reduce development time, and focus the evaluation process (15). There is a need to assess whether CCC programs already use evidence-based interventions and encourage those that do not to consider them when planning activities. The objective of this content review is to identify whether programs reference evidence-based screening recommendations and interventions for CRC in their CCC plans, with a focus on prevention and early detection.

## Methods

In January 2008, we reviewed all CCC plans (n = 55) posted on the Cancer Control PLANET Web site (http://cancercontrolplanet.cancer.gov). This Web site contains all published CCC plans and is regularly updated as new plans are disseminated. A list of search terms to identify evidence-based content was compiled on the basis of a content review of topics in CCC plans (16). Other search terms likely to identify evidence-based content were included, as were names of organizations involved in reviewing or disseminating evidence-based information on cancer prevention. The feasibility of using key terms for the entire review was determined by using the terms to test a select number of plans. We excluded terms that were either too ambiguous or unlikely to yield evidence-based content related to CRC prevention and early detection.

We used the search feature in Adobe Acrobat Reader version 8 (Adobe Systems Inc, San Jose, California) to search each cancer plan for colorectal or colon cancer content. The paragraph related to the term was reviewed for specific key terms to potentially identify evidence-based content (Table 1). An abstraction tool to facilitate review was created in Microsoft Excel (Microsoft Corporation, Redmond, Washington). Content items were copied and then coded within this tool. Items were included if they referred to screening, interventions, or evidence-based sources or programs (including how to build or develop the evidence base). Only items specific to CRC prevention and early detection were abstracted. Names of organizations (eg, American Cancer Society) were only included if they were in the context of screening guidelines, evidence-based interventions, research projects, or the development of interventions. Therefore, items that only identified the organization were excluded (eg, name of person affiliated with the organization). In some instances, the key terms identified content that was clearly not evidence-based or was too ambiguous to classify. When this situation was encountered, the content was not abstracted after determining that it could not be classified as evidence-based.

Key terms included the following exact phrases or words: *best practice(s), effective/effectiveness, established, evaluated intervention trial, evidence-base(d)/evidence base(d), evolving science, guideline(s), proven, research into practice, research-tested, science-based, scientific evidence, translation of research,* and *tested*. The following organizations and sources of evidence-based recommendations and interventions (documents and Web sites) were included as key terms: American Cancer Society (ACS), Cochrane Reviews, the Task Force on Community Preventive Services' *Guide to Community Preventive Services (Community Guide)*, the National Cancer Institute's Physician Data Query (NCI's PDQ), the Agency for Healthcare Research and Quality (AHRQ), Put Prevention into Practice (PPIP), NCI's and Substance Abuse and Mental Health Services Administration's (SAMHSA) Research Tested Intervention Programs (RTIPs), and United States Preventive Services Task Force (USPSTF) recommendations — *Guide to Clinical Preventive Services*. If multiple key terms identified the same content, each additional key term was also recorded.

The context of each key term was evaluated for whether it appeared in background information; a goal, objective, or strategy; an activity outside of a goal, objective, or strategy; a recommendation that will be promoted; or other. The section or chapter of the cancer plan where the term was located was categorized according to the following sections along the cancer control continuum: primary prevention, secondary prevention or early detection, treatment, palliation or end-of-life care, and survivorship. Other categories were based on where the content was found in the plan, including executive summary, introduction, conclusion, health disparities, and other. If recorded as "other," the specific title of the section or chapter was recorded. Because not all cancer plans were organized along the cancer control continuum, the "other" category often included chapters specifically addressing CRC. Direct mention of the following evidence-based cancer prevention recommendations and sources were noted and categorized as follows: *Community Guide*, Cochrane Reviews, NCI's PDQ, USPSTF, ACS, PPIP, and RTIPs. The year that the cancer plan was published (identified as a publication date or dated letter from a state official), Cochrane Reviews, NCI's PDQ, USPSTF, ACS, PPIP, and RTIPs. The year that the cancer plan was published (identified as a publication date or dated letter from a state official) was recorded. In the absence of a publication date, the first year of implementation of the cancer plan was used. The evidence-based content in the CCC plans was further evaluated and categorized according to whether it referred to 1) select evidence-based screening/early detection guidelines (ie, published by ACS or USPSTF or listed in NCI's PDQ) or 2) an evidence-based program or intervention in the community. The second category included interventions that were being proposed, planned, developed, adapted; were currently in use; or had been used. Some evidence-based content appeared as background or reference material only and was categorized as such. CCC plans could have content in all categories.

Two separate analyses were conducted. The first analysis examined the evidence-based content itself to describe the type of content that appeared in the CCC plans, and the second was a program-level analysis to identify the number of programs referring to specific types of evidence-based interventions or screening guidelines. The latter analysis was conducted to identify gaps in addressing evidence-based interventions in current CCC plans. All descriptive analyses were conducted using SAS version 9 (SAS Institute, Inc, Cary, North Carolina) after importing the abstracted and coded content from Excel.

## Results

Fifty-four CCC plans (98%) had content that mentioned an evidence-based recommendation or intervention surrounding CRC prevention and early detection or indicated they intended to use an evidence base when developing programs and activities. The 1 CCC plan that did not include identifiable evidence-based content was excluded from further analysis. Therefore, the denominator for all program analyses was 54.

Plans were released between 2000 and 2007. Release years could not be identified for 2 states. Of 52 CCC plans with evidence-based content and a known publication or release date, 2005 was the most common publication or release year (n = 19). Sixty-five percent (n = 34) of the 52 CCC plans had a publication or release date of 2005 or later.

Overall, we abstracted 186 evidence-based content items related to CRC prevention and early detection from 54 CCC plans. Nearly 54% (n = 100) of content items were found in sections of the plans with titles specific to secondary prevention or early detection (data not shown), 20% (n = 37) were found in sections with titles specific to CRC, 1.6% (n = 3) were found in primary prevention, and the remaining 25% of content (n = 46) were found in sections such as the executive summary, health disparities, appendices, or plan-specific categories.

Six key terms — *evaluated intervention trial, evolving science, research into practice, research-tested, tested,* and *translation of research* — did not yield any evidence-based content for prevention and early detection of CRC (Table 1). Multiple key terms identifying the same evidence-based content were encountered in 37% of abstracted content (data not shown). Commonly encountered key terms appearing together were *guidelines* and *ACS*; *guidelines*, *ACS*, and *USPSTF*; and *ACS* and *USPSTF*. Among CCC programs, 34 referred to ACS as a key term when discussing evidence-based interventions (notably screening guidelines) for CRC, 30 used the term *guidelines*, 22 used the term *evidence-based*, 22 referred to the USPSTF, and 18 used the term *effective* or *effectiveness* (Table 1).

Nearly 65% (n = 35) of CCC programs had evidence-based content appearing in a goal, objective, or strategy, while approximately 76% (n = 41) had content that appeared as background information in a chapter or section (Table 2). Nearly 15% (n = 8) of programs had evidence-based content in reference to an activity appearing outside of a goal, objective, or strategy, while slightly more than 5% (n = 3) included it as part of a recommendation. One plan included evidence-based content in a context outside of these classifications as a statement of a vision. More than 57% (n = 31) of CCC programs referred to ACS guidelines, nearly 41% (n = 22) referred to the USPSTF, and approximately 11% (n = 6) referred to the *Community Guide*. Few programs mentioned RTIPs (n = 1), NCI's PDQ (n = 4), Cochrane Reviews (n = 2), or PPIP (n = 2) in reference to evidence-based interventions for CRC prevention.

Nearly 53% of identified content (98 of 186) referred to evidence-based screening guidelines for CRC (notably ACS, USPSTF, NCI's PDQ). Approximately 30% (n = 56) described an evidence-based intervention or program intended for use or that had been used in the community, and approximately 17% (n = 32) referred to evidence-based interventions as background information or reference material (Table 3). Nearly 78% of CCC programs (42 of 54) referred directly to evidence-based screening recommendations for CRC in their CCC plans; almost 60% (n = 32) described evidence-based interventions or programs in use, being proposed, or in development for their program area; and nearly 30% (n = 16) also referred to evidence-based interventions in their background information. These categories are not mutually exclusive. In 4 CCC plans, evidence-based interventions were described in the context of background information; there were no references in the body of the plan to evidence-based CRC interventions the programs planned to implement. References to evidence-based interventions in 17 plans were limited to screening guidelines. One program referred to both screening guidelines and evidence-based interventions to increase screening, but the reference also was in the context of background information.

Table 4 provides some examples of content items that appeared in CCC plans about evidence-based interventions for CRC prevention and how it appeared in the plan (goal, objective, or strategy; background information; activity; recommendation). Many content items were encountered. Examples include evidence-based screening guidelines (either referencing these or using them), community-based research projects being conducted, proposed community-based interventions, and intentions to use the evidence base when developing future projects.

## Discussion

For nearly all CCC plans, the evidence-based content included at least 1 reference to CRC prevention and early detection. We encountered varying degrees of levels of evidence during this content analysis, ranging from plans with the most objective evidence (evidence-based guidelines and systematic reviews) to those with more subjective evidence (evidence-informed programs and program evaluation) (15). Nearly 60% of CCC plans describe a program or intervention in their respective areas that may be considered evidence-based. Evidence-based interventions are those with proven effectiveness within the populations and settings in which the interventions were studied (15).

Unlike breast cancer screening, which has established interventions to increase screening, few published evidence-based interventions exist for the prevention and early detection of CRC (26,27). According to the *Community Guide*, compared with breast cancer screening, CRC screening has fewer recommended interventions and more interventions with "insufficient evidence" (26). Research-tested interventions are defined by NCI as those tested in a peer-reviewed and funded research study (15) and are a limited subset of evidence-based interventions. Since CCC programs have a smaller number of evidence-based resources and interventions available for CRC, some rely exclusively on practice-based evidence when developing or revising their CCC plans. Few plans explicitly referenced RTIPs, Cochrane Reviews, or PPIP. This finding reflects the paucity of information available on evidence-based interventions to increase CRC screening. For example, only 5 research studies were posted on the RTIPs site (27) for CRC screening, compared with 13 for breast cancer screening.

Another challenge programs face when they search for evidence-based interventions is finding interventions that will work in their locales (28). Practice-based evidence derives from experience within practice settings and addresses issues around external validity and local realities in which a program operates (29). The lack of readily available practice-based evidence also makes it difficult to locate appropriate interventions. To help remedy this situation, CDC supports ongoing projects to identify effective, culturally sensitive intervention strategies to promote CRC screening in communities (30). These projects are being developed and tested in varied populations and settings, including the medically underserved in Appalachia, the lower Rio Grande Valley in Texas, and various metropolitan areas and communities of the United States. Results from these projects will contribute to the body of research evidence and fill in gaps in the *Community Guide*.

Even though CCC programs frequently referred to evidence-based CRC interventions in their plans, specific information regarding the identification and implementation of interventions was not obvious in the content. For example, interventions recommended by the *Community Guide* (26), such as client reminders or small media, that increase CRC screening were mentioned in a number of CCC plans. However, only 6 CCC plans clearly identified the *Community Guide* when discussing interventions. These findings may indicate a need for training on how to find and select culturally sensitive evidence-based interventions and an efficient mechanism to share information on evidence-based interventions with practitioners (28).

### Limitations

One major limitation of this content analysis was that some CCC plans grouped "screenable" cancers together, most often breast, colorectal, and cervical cancers. This grouping made identifying content related specifically to CRC difficult and required entire sections on all "screenable" cancers to be read to find evidence-based content.

Another limitation was that evidence-based content items that did not specifically include the key terms were not identified in this analysis. Because it was not always clear from the content in the CCC plans whether a program or intervention was evidence-based, some items may have been included that do not fall into this context. This determination was left to the judgment of a single reviewer. CCC programs were not contacted to verify their evidence-based content. Content analyses are subjective and are vulnerable to reviewer bias and coding inconsistencies when manually coded. Other documents, such as work plans, which may describe evidence-based intervention activities being conducted or implemented, were not reviewed as part of this study. Furthermore, some key terms used, such as *guidelines* and *effective/effectiveness*, did not always yield evidence-based content related to CRC. This content was either not abstracted or was removed after later determination.

Lack of common terminology surrounding dissemination and implementation may have contributed to the lack of consistent nomenclature in the plans. Rabin et al reported that, because research regarding program dissemination and implementation has origins in multiple disciplines, there are inconsistencies in the use and definitions of terms and concepts (31). The glossary they developed for dissemination and implementation research in health provides a starting point for researchers and program people to be able to speak a common language.

### Conclusion

Substantial illness and death from CRC still exist even though screening is effective. As public health professionals, we need to find better ways to disseminate and apply cost-effective, culturally sensitive interventions that promote CRC screening. In some cases, there may be a need to convince communities to accept evidence-based interventions and abandon ineffective practices. In situations where the evidence base is lacking, we need to evaluate existing programs or build on promising practices to assist in developing the evidence base. To help CCC programs reduce the burden of CRC among the populations they serve, we recommend the following: train programs to locate culturally sensitive, evidence-based interventions (15,32); encourage them to conduct and publish intervention studies (33-35); promote the use of consistent common language around intervention science (31); and encourage the research community to include more practice-based evidence in evidence-based databases (29,36,37).

## Figures and Tables

**Table 1:** Evidence-Based Content for Colorectal Cancer Identified in State, Tribal Governments and Organizations, Territories, and Pacific Island Jurisdictions' Comprehensive Cancer Control Plans (n = 54) by Search Term

**Search Term**	No. of Plans (%) **a**
American Cancer Society	34 (63.0)
Guideline(s)	30 (55.6)
Evidence-base(d)/evidence base(d)	22 (40.7)
United States Preventive Services Task Force *Guide to Clinical Preventive Services*	22 (40.7)
Effective/effectiveness	18 (33.3)
*Guide to Community Preventive Services/Community Guide*	6 (11.1)
Best practice(s)	5 (9.3)
Established	5 (9.3)
Proven	4 (7.4)
National Cancer Institute's Physician Data Query	4 (7.4)
Cochrane Reviews	2 (3.7)
Agency for Healthcare Research and Quality's Put Prevention Into Practice	2 (3.7)
National Cancer Institute's and Substance Abuse and Mental Health Services Administration's Research Tested Intervention Programs	1 (1.9)
Scientific evidence	1 (1.9)
Science-based	1 (1.9)
Evaluated intervention trial	0
Evolving science	0
Research into practice	0
Research-tested	0
Tested	0
Translation of research	0

a Percentages do not total to 100% because plans may have multiple content items identified through multiple search terms.

**Table 2:** Evidence-Based Content for Colorectal Cancer Identified in State, Tribal Governments and Organizations, Territories, and Pacific Island Jurisdictions' Comprehensive Cancer Control Plans (n = 54), by Content Type and Reference Source

**Content**	No. of Plans (%) **a**
**Type**	
Background information	41 (75.9)
Goals/objectives/strategies	35 (64.8)
Activity	8 (14.8)
Recommendation	3 (5.6)
Other	1 (1.9)
**Reference source**	
American Cancer Society guidelines	31 (57.4)
United States Preventive Services Task Force	22 (40.7)
*Guide to Community Preventive Services/Community Guide*	6 (11.1)
National Cancer Institute's Physician Data Query	4 (7.4)
Cochrane Reviews	2 (3.7)
Agency for Healthcare Research and Quality's Put Prevention into Practice	2 (3.7)
National Cancer Institute's and Substance Abuse and Mental Health Services Administration's Research Tested Intervention Programs	1 (1.9)

a Percentages do not total to 100% because plans may have multiple content items identified through multiple search terms.

**Table 3:** Grouping of Evidence-Based Content for Colorectal Cancer Identified in State, Tribal Governments and Organizations, Territories, and Pacific Island Jurisdictions' Comprehensive Cancer Control Plans, Content-Level and Program-Level Analysis

**Evidence-Based Content Category**	Content-Level (n = 186) Frequency (%)^a^	Program-Level (n = 54) Frequency (%)^a^
Evidence-based screening guidelines	98 (52.7)	42 (77.8)
Developing/proposing/adapting/using evidence-based interventions (including evidence-informed programs)	56 (30.1)	32 (59.3)
Refer to evidence-based interventions, but as background information	32 (17.2)	16 (29.6)

a Percentages do not total to 100% because plans may have multiple content items identified through multiple search terms.

**Table 4:** Examples of Evidence-Based Content Items for Colorectal Cancer Identified in States, Tribal Governments and Organizations, Territories, and Pacific Island Jurisdictions' Comprehensive Cancer Control Plans

**Example**	Content Type/Appearance
"By 2010, increase the proportion of adults aged 50 and older to 22 percent for FOBT and 41 percent for sigmoidoscopy or colonoscopy who have received these colorectal cancer screening consistent with ACS and USPSTF guidelines." (17)	Goals/objectives/strategies
"Assist health care systems in using practice-based tools and techniques that will ensure cancer early detection services are discussed/provided to all eligible patients, according to recommended guidelines." (18)	Goals/objectives/strategies
" . . . [D]evelop and support evidence-based, culturally sensitive public awareness campaigns that focus on the importance of colorectal cancer screening, prevention, and early detection through media, community outreach, and through a collaboration among health care providers and community and voluntary organizations . . . " (19)	Goals/objectives/strategies
"The CDC *Guide to Community Preventive Services* provides evidence-based interventions that community leaders, policy makers, and decision makers can apply to increase the utilization of colorectal cancer screening methods." (20)	Background information
"The US Preventive Services Task Force (USPSTF) strongly recommends men and women 50 years of age or older be screened for colorectal cancer. They found that several screening methods are effective in reducing mortality from colorectal cancer." (21)	Background information
" . . . [T]he American Cancer Society (ACS) recommends screening average-risk asymptomatic people for colorectal cancer to begin at age 50. According to the ACS guidelines . . . " (22)	Background information
"Juntos en la Salud is a 5-year behavioral and cancer screening project funded by the American Cancer Society, which aims to assess the effectiveness of improving breast, cervical, and colorectal cancer screening rates and general lifestyle prevention behaviors among low-income Latinas through the development of social support groups with lay health educators. " (23)	Activity
"In terms of research, Harvard Center for Cancer Prevention received a Targeted Intervention Opportunity Grant (TIOG) from the American Cancer Society. The results of the research conducted with this grant, 'Improving Colorectal Cancer Screening by Targeting Office Systems in Primary Care Practices: Disseminating Research Results Into Clinical Practice,' were recently published in the Archives of Internal Medicine." (24)	Activity
"Monitor emerging science. . . . Published research on public health interventions should also be monitored to identify effective approaches for increasing screening rates particularly among populations with lower screening rates." (25)	Recommendation

Abbreviations: FOBT, fecal occult blood test; ACS, American Cancer Society; USPSTF, United States Preventive Services Task Force; CDC, Centers for Disease Control and Prevention.
